# The Validity of the SEEV Model as a Process Measure of Situation Awareness: The Example of a Simulated Endotracheal Intubation

**DOI:** 10.1177/0018720821991651

**Published:** 2021-02-17

**Authors:** Tobias Grundgeiger, Anna Hohm, Annabell Michalek, Timo Egenolf, Christian Markus, Oliver Happel

**Affiliations:** 1 9190 Institute Human-Computer-Media, Julius-Maximilians-Universität Würzburg, Würzburg, Germany; 2 27207 University Hospital Würzburg, Würzburg, Germany

**Keywords:** eye tracking, laryngoscopy, situation awareness measure, visual attention, SEEV model

## Abstract

**Objective:**

In the context of anesthesiology, we investigated whether the salience effort expectancy value (SEEV) model fit is associated with situation awareness and perception scores.

**Background:**

The distribution of visual attention is important for situation awareness—that is, understanding what is going on—in safety-critical domains. Although the SEEV model has been suggested as a process situation awareness measure, the validity of the model as a predictor of situation awareness has not been tested.

**Method:**

In a medical simulation, 31 senior and 30 junior anesthesiologists wore a mobile eye tracker and induced general anesthesia into a simulated patient. When inserting a breathing tube into the mannequin’s trachea (endotracheal intubation), the scenario included several clinically relevant events for situation awareness and general events in the environment. Both were assessed using direct awareness measures.

**Results:**

The overall SEEV model fit was good with no difference between junior and senior anesthesiologists. Overall, the situation awareness scores were low. As expected, the SEEV model fits showed significant positive correlations with situation awareness level 1 scores.

**Conclusion:**

The SEEV model seems to be suitable as a process situation awareness measure to predict and investigate the perception of changes in the environment (situation awareness level 1). The situation awareness scores indicated that anesthesiologists seem not to perceive the environment well during endotracheal intubation.

**Application:**

The SEEV model fit can be used to capture and assess situation awareness level 1. During endotracheal intubation, anesthesiologists should be supported by technology or staff to notice changes in the environment.

## Introduction

Situation awareness—knowing “what is going on”—is important for safe operation in safety-critical domains such as anesthesiology. For example, during the induction of general anesthesia, the patient’s status can change quickly, and good situation awareness is important for patient safety (Schulz et al., 2013, 2016). Although research has highlighted the importance of visual attention distribution for situation awareness in aviation (e.g., Stein, 1992; Ziv, 2016), plant control (e.g., Mumaw et al., 2000), and anesthesiology (e.g., Gaba et al., 1995; Schulz et al., 2011), visual attention distribution has frequently only been analyzed descriptively (i.e., percentage of fixations or dwell time on several objects; for a review in healthcare, see Grundgeiger et al., 2015). The salience effort expectancy value (SEEV) model of visual attention provides a theoretically founded way of investigating attention distribution (Wickens, 2015; Wickens et al., 2003). Wickens et al. (2008) suggested that the SEEV model fit should correspond to situation awareness level 1 (perception or noticing of elements in the environment). We tested this suggestion in the context of anesthesiologists inducing general anesthesia in a simulated patient by correlating the SEEV model fit with situation awareness scores.

### Eye Tracking Metrics as a Situation Awareness Measure

Situation awareness can be described as the perception or noticing of elements in the environment (SA level 1), comprehension of their meaning (SA level 2), and understanding of future implications (SA level 3; e.g., Endsley, 1995; Wright et al., 2004). The Situation Awareness Global Assessment Technique (SAGAT, Endsley, 1995) is the most common technique used to assess situation awareness (de Winter et al., 2019; Endsley, 2021); however, there is an ongoing debate about the advantages and disadvantages of SAGAT (de Winter, 2020; de Winter et al., 2019; Endsley, 2015, 2020; Stanton et al., 2017). One alternative measure is the use of eye tracking metrics, which is a so-called process measure of situation awareness (i.e., an ongoing measure rather than measuring at a specific point in time; Salmon et al., 2006). Endsley (2021) summarized that eye tracking has the advantage of being objective, and eye tracking includes information about order and duration of attention distribution, yet the technique does not consider auditory information, and overall there is little research that supports the validity of eye tracking as a process measure of situation awareness.

In a recent review on physiological measurements of situation awareness, Zhang et al. (in press) found 16 papers that reported eye tracking metrics and situation awareness scores but only a limited number of papers specifically addressed the association between conscious eye tracking metrics (i.e., dwell times, number of fixations, etc.) and situation awareness scores. Moore and Gugerty (2010) used various eye tracking metrics of certified air traffic controllers in a simulated setting to predict a direct situation awareness measure. Moore and Gugerty (2010) observed that the percentage of time fixating on a specific aircraft could explain 7% and 9% of the unique variance in SA level 1 and a composite overall SAGAT score (SA levels 1–3), respectively. In the context of healthcare, Law et al. (2020) manipulated the position of the patient monitor during a simulated neonatal resuscitation. As a secondary analysis, Law et al. (2020) reported a nonsignificant correlation of *r_s_* = .39 (*p* = .07) between a composite SAGAT score (SA levels 1–3) with the percentage of visual attention on the patient monitor. In the context of tripping hazards on construction sites, Hasanzadeh et al. (2016) correlated several eye tracking metrics of undergraduates working on a construction site task with a subjective situation awareness measure (Situational Awareness Rating Technique, SART) and observed a negative association of dwell time and fixation count with situation awareness. However, the area of interest (AOI) description was limited and the negative association was not discussed. In a further analysis, Hasanzadeh et al. (2018) reported that the four participants with high SART scores payed more attention to feedforward-information and environmental-related AOI than seven participants with low SART scores. Finally, in a simple gage monitoring laboratory-based task, de Winter et al. (2019) observed a correlation of 0.31 between the percentage of glances at a specific task-relevant AOI and a task performance score; however, a freeze-probe score—percentage of glances correlation was only 0.10 (de Winter, personal communication).

By investigating the correlation of the SEEV model fit and SAGAT score, we contribute to and extend the above research in several ways. First, previous research with actual domain experts (e.g., Law et al., 2020; Moore & Gugerty, 2010) was limited in relation to sample size (*N* = 11–29). The present sample includes 61 qualified anesthesiologists. Second, previous research considered only a single AOI (e.g., one aircraft’s flight strip and radarscope icon or vital sign monitor) for the analysis of eye tracking metrics. In the SEEV model, the value of an AOI in relation to the main goals of an operator is assessed and combined. This way, the SEEV model enables the inclusion of as many AOI as needed in one combined analysis. Third, considering only one AOI limits any situation awareness question related to this AOI (Moore & Gugerty, 2010). Otherwise, the plausibility of associating situation awareness questions not relating to the AOI under study may be questioned. Fourth, the SEEV model is built on the basis of cognitive science and engineering research on visual attention and therefore provides a theoretical foundation to investigate the relation between attention distribution and situation awareness.

### The Salience Effort Expectancy Value (SEEV) Model

The SEEV model integrates the factors *salience*, *effort*, *expectancy*, and *value* to predict the allocation of overt visual attention in a supervisory control task (for reviews, see Steelman et al., 2017; Wickens, 2015). The factor salience denotes the features of an AOI that possibly will attract attention. The factor effort refers to the exertion required to access information such as head turns. Salience and effort are both bottom-up factors because both depend on the environment. The factor expectancy refers to the bandwidth (i.e., rate of information change) within an AOI. The factor expectancy combines the rate of information change and the fact that qualified operators have an expectation about the event rate in a specific AOI. Finally, the factor value refers to the importance of an AOI to the main goal of an anesthesiologist. Expectancy and value are top-down factors and depend mainly on the knowledge of the operator.

When using the SEEV model as an analytic equation model (Wickens et al., 2003), the factors salience, effort, expectancy, and value are combined to predict the so-called percentage dwell time (PDT). The PDT is the relative proportion of time for which attention is allocated to a specific AOI such as the patient. In the so-called EV version of the SEEV model, the coefficients of the bottom-up factors salience and effort are set to zero, leaving only the two top-down factors, expectancy and value, in the model. Wickens (2015) and Wickens et al. (2008) noted that the EV model can be used to assess “optimal” attention distribution, because in the EV version, the allocation of attention is driven only by factors that should guide the attention of experts. The EV model provides, therefore, a gold standard against which the observed dwell time distributions can be compared. In addition, empirical studies showed that including the factors salience and effort (Steelman et al., 2011) or only the factor effort (Grundgeiger et al., 2020; Wickens et al., 2008) did not increase the model fit of experts.

Wickens et al. (2008) connected the SEEV model and situation awareness by suggesting the so-called Attention-Situation Awareness model. In short, the model incorporates two modules. The attention module includes the SEEV model, which considers the allocation of overt visual attention and therefore describes the information seeking behavior of an operator. The attended information is integrated by the belief module to update the level of situation awareness. High situation awareness enables the operator to make informed decisions (rather than relying on outdated information or pre-existing knowledge) but also enables the capacity to guide attention to events of high value. In relation to situation awareness levels (Endsley, 1995), the attention module represents level 1 (perception of the environment), and the belief module represents level 2 and 3 (integration of information, anticipation of the future; Wickens et al., 2008). To the best of our knowledge, the full Attention-Situation Awareness model has so far been used only in a sensitivity analysis in which the parameters were manipulated in a computational simulation (Wickens et al., 2008).

### The Present Study

In the present study, junior and senior anesthesiologists wore a mobile eye tracker and induced general anesthesia in a simulated patient (manikin). For situation awareness, we focused on the most critical and demanding phase, which is the endotracheal intubation (Weinger et al., 2000). To measure SA level 1, we implemented several events into this phase that were relevant for the immediate management of the patient such as a sudden drop in patient heart rate from 74 to 51 bpm. In addition, following Cooper et al. (2010), we implemented further events that were not relevant for immediate patient safety to investigate the general perception of environmental events, such as an operating light switching off. Immediately after the intubation was finished and the anesthesiologists were led outside the simulation room, they completed a questionnaire that included questions about clinical variables and events and environmental events (Table 1) to assess the awareness of the anesthesiologist during the phase of endotracheal intubation. Finally, we asked one open-ended question addressing SA level 2 and one question addressing SA level 3.

**Table 1:** The 13 Questions Addressing Environmental Perception, Situation Awareness (SA) Level 1–3

#	Question	Perception of	Correct
1	Did the display of the wall-mounted clock change from analog to digital during the laryngoscopy?	Environment	Yes
2	Did somebody enter the room during the laryngoscopy?	Environment	No
3	Did the nurse switch the phone from the right to the left ear during the phone call?	Environment	No
4	Do you know the content of the phone call? Please provide a short description	Environment	Shift change
5	Did the nurse wear a watch during the laryngoscopy?	Environment	Yes
6	Was the syringe for blocking the cuff attached to the endotracheal tube during the endotracheal intubation?	Environment	No
7	Did the anesthetic machine give a warning or alarm during the laryngoscopy?	SA level 1	Yes
8	Was one of the operating lights switched off during the laryngoscopy?	Environment	Yes
9	What was the blood pressure of the patient during the laryngoscopy? Please provide an exact value	SA level 1	116 systolic^a^ 46 diastolic^a^
10	What was the heart rate of the patient during the laryngoscopy? Please provide an exact value	SA level 1	51 bpm^a^
11	What was the oxygen saturation of the patient during the laryngoscopy? Please provide an exact value	SA level 1	>=97%
12	How would you describe the actual status of the patient?	SA level 2	-
13	Where do you see the patient’s status in the next few minutes? Is there anything that you would pay attention to in the next few minutes?	SA level 3	-

*Note*. For all closed questions, participants provided answers using a 4-point scale (sure yes, yes, no, sure no) but for the analysis, a eifferentiation was made only between yes and no.

^a^±10% margin to determine whether an answer was right or wrong.

We calculated the EV model fit for each individual anesthesiologist by correlating the observed PDT based on the eye tracking recordings of the whole procedure of inducing general anesthesia with the predicted PDT by the EV model. Next, we correlated the individual model fit with the individual perception and situation awareness scores. Based on the Attention-Situation Awareness model, we expected a positive correlation between the model fit with the different perception scores (global, SA level 1, and environmental). Because of the specific focus on the laryngoscopy for endotracheal intubation, we also separately assessed the correlation between the model fit for laryngoscopy phase with perception scores. Finally, we compared the individual model fit of the participants who were scored to have overall situation awareness based on the assessment of a blinded subject-matter expert reviewing the answers to all questions (including SA levels 2 and 3) with the other participants. We expected that participants with situation awareness would show a better mean model fit compared to the other participants.

## Method

### Participants

A total of 67 anesthesiologists of the Department of Anaesthesia and Critical Care at the University Hospital Würzburg participated. Due to technical failure (3), failure in the experimental procedure (1), poor eye tracking quality (1), or no consent for eye tracking (1), six participants were excluded from the analysis. Junior anesthesiologists had a mean age of *M* = 30 years (*SD* = 4; gender *f*/*m*: 19/11) and a working experience of *M* = 1.26 years (*SD* = 1.54). Senior anesthesiologists had a mean age of *M* = 37 years (*SD* = 3; gender *f*/*m*: 12/19) and a working experience of *M* = 8.69 years (*SD* = 2.55). The difference in gender distribution was not significant (Fisher’s Exact test, *p* = .074) but the descriptive difference is representative for anesthesiologists’ gender distribution in Germany (van den Bussche et al., 2019). This research complied with the Declaration of Helsinki and was approved by the Institutional Review Board at the University of Würzburg. Informed written consent was obtained from each participant.

### Design

An independent variable was experience (junior, senior). An anesthesiologist with a special 5-year training in anesthesiology (i.e., consultant) was considered to be a senior anesthesiologist. Half of the participants used a video-based device for laryngoscopy and the other half used a so-called Macintosh blade which is used as the standard device for a direct laryngoscopy. However, this comparison is not the focus of the present analyses.

As dependent variable, we measured the perception of different events in the environment. We calculated a SA level 1 score that included only events that were immediately relevant for managing the patient. We calculated an environment perception score that included events that happened in the simulation room environment but were not critical for immediate patient safety. We combined the SA level 1 score and the environment perception score in a global situation perception score. In addition, we used one question each to address situation awareness levels 2 and 3. For an overall situation awareness score, a blinded subject matter expert combined all SA level questions and classified participants in having overall situation awareness or not (see method section).

### Procedure and Material

Participants put on the mobile eye tracker (SMI Eye Tracking Glasses, Teltow, Germany). The first four phases of the procedure (checking case history, precheck of equipment, preoxygenation and induction, mask ventilation and muscle relaxation) were uneventful. During the fifth phase (laryngoscopy), several events happened that were either triggered by the experimenter in the control room (i.e., operating room light switched off) or by the nurse (i.e., put on watch) that were used to measure the perception of the environment and SA level 1. For a detailed description of the content and timing on the events, see supplemental material.

After the blade of the laryngoscopy device was removed from the mannequin’s mouth (i.e., end of laryngoscopy), the experimenter entered the simulation room, indicated that the scenario was over, and guided the participant out of the simulation room. Approximately 10–15 seconds after the end of the scenario, the participant filled in a questionnaire including 13 questions about the laryngoscopy phase. The questions are provided in Table 1 and an illustration of the respective events is provided in supplemental Figure S1. Because we focused on the laryngoscopy, we did not conduct a goal directed task analysis but subject matter experts (authors CM and OH) developed situation awareness questions considering the anesthesiologist’s goals for the induction of general anesthesia (King & Weavind, 2017; Weinger & Slagle, 2002). For the environmental perception, we tested to what extent the anesthesiologist perceived the changes in the environment.

Finally, participants provided demographic data. The procedure was rehearsed several times to ensure the exact timing of events and was tested with a pilot participant. All participants received a five Euro cafeteria voucher. The average scenario length was 7:59 min (no differences between experience groups) and the whole experimental session lasted approximately 30 min.

### Calculation of Perception Scores

For the first 11 questions, each correctly answered question scored one point. If no answer was given, we did not consider the answer in the analysis because it is unclear whether the participants did not know the answer or accidentally skipped a question (7 instances, 96% of the answers). If only one part of a question was addressed or participants indicated with a symbol such as a question mark that they did not know the answer, we assigned zero points (12 instances, 1.64% of the answers).

We calculated the scores based on the relative proportion of correctly answered questions. We calculated the global situation perception score based on eleven questions, the environment perception score based on the seven questions that related to events in the environment but were not immediately relevant for patient safety, and a SA level 1 score based on the clinically relevant questions (four questions with five items). Finally, author OH was blinded in relation to any participants’ demographics and assessed the answers to all questions and the two open questions regarding situation awareness levels 2 and 3 and assigned an overall situation awareness score in relation to patient clinical status of either 0 or 1. OH did not receive any information about the participants such as experience level. For 19 participants, question 8 had a misleading formulation (i.e., we did not explicitly ask whether an operating room light but any light in the room switched off) and was not considered in the analysis for these participants.

### Calculation of Model

We provide a brief summary of the AOI analysis, the model parameters, and the model calculations which were based on Wickens and colleagues (Wickens et al., 2001, Wickens et al., 2003). For a detailed description and example calculations, see Grundgeiger et al. (2020).

First, based on previous work (Grundgeiger et al., 2017; Schulz et al., 2011), we manually assigned each fixation to an AOI and grouped the single AOI in four semantically related groups (patient, monitoring equipment, documentation, medication + general equipment) and a “Not included in analysis” group (see note of Table 2 for assignment of single AOI and Supplemental Figure S2). Second, based on the literature (King & Weavind, 2017; Weinger & Slagle, 2002), the two main goals for the induction of general anesthesia can be described as (1) rapidly, safely, and pleasantly producing amnesia, analgesia, akinesia, and autonomic and sensory block while (2) maintaining hemodynamic stability and sufficient ventilation. Third, considering the main goals for the induction of general anesthesia, three domain experts assigned the required model parameters. Fourth, we calculated the predicted PDT for the AOI groups (for more information, see supplemental Tables S1 and S2).

**Table 2:** Observed Percentage Dwell Time on Different Groups of Area of Interest (AOI Groups) Separated by Phase and Experience

AOI Groups	Phase 1 Case History		Phase 2 Precheck		Phase 3 Preoxygenation and Induction		Phase 4 Mask Ventilation and Relaxation		Phase 5 Laryngoscopy	
	Junior	Senior	Junior	Senior	Junior	Senior	Junior	Senior	Junior	Senior
Patient	27.86 (17.01)	23.30 (11.7)	14.47 (10.31)	13.46 (8.78)	53.98 (16.59)	48.35 (15.73)	44.85 (16.72)	50.22 (17.71)	92.43 (5.86)	90.90 (7.78)
Monitoring equipment	1.66 (4.51)	1.15 (2.08)	25.17 (10.82)	21.95 (12.25)	16.46 (10.33)	16.5 (10.89)	33.93 (13.02)	23.32 (15.45)	0.53 (1.25)	0.64 (2.76)
Documentation	59.73 (17.59)	59.97 (14.51)	1.07 (3.03)	2.94 (5.91)	1.61 (6.16)	1.07 (2.16)	0.10 (0.34)	0.28 (0.71)	0	0
Medication + general equipment	3.22 (4.42)	4.28 (5.75)	48.77 (18.05)	48.37 (15.72)	18.10 (10.32)	21.26 (9.31)	13.93 (7.22)	13.63 (7.84)	5.96 (5.11)	6.25 (5.07)
Not included in EV analysis	7.52 (4.56)	11.30 (6.3)	10.52 (7.15)	13.29 (7.39)	9.86 (7.13)	12.83 (7.7)	7.19 (5.42)	12.55 (12.07)	1.09 (1.36)	2.20 (3.39)

*Note.* Values indicate *M* (*SD*). AOI group patient included patient’s head, patient’s thorax, IV access, face mask (phase 3–5, only), patient’s mouth, video monitor of laryngoscope (phase 5, only), and patient’s arm. AOI group monitoring equipment included anesthesia machine, patient monitor, changing settings on patient monitor, changing settings on anesthesia machine, and clock. AOI group documentation included patient record and anesthesia chart. AOI medication + general equipment group included nurse’s hands, infusion, face mask (phase 1–2, only), respiratory tube, application of drugs, anesthesia trolley, video monitor of laryngoscope (phase 1–4, only), and monitoring cable. Not included in EV analysis were nurse’s head, fixation due to movement in room, floor, walls, content of front pocket, IV pumps not in use, desk, entrance door to simulation, and not classified (i.e., sink, etc.).

As in previous research (Wickens et al., 2003, 2008), we used Pearson correlations (*r*) to correlate the predicted PDTs according to the model with the overall observed PDT to calculate the model fit. Furthermore, the predicted PDT by the model were correlated with the observed PDTs of each participant. We calculated Spearman’s rho (*r_s_*) correlations to assess the association of model fits and the situation awareness scores. The statistical analysis was conducted with IBM SPSS Statistics (Version 25.0. Armonk, NY: IBM Corp.). Alpha was set at .05.

## Results

### Perception and Situation Awareness Scores

Overall, the situation perception scores and situation awareness scores were not high. Initially, we collected data of 50 participants and noted the general low scores. To test whether the scenario was too demanding to notice changes in relation to SA level 1 and build overall situation awareness, we ran the scenario with an additional twelve participants (six junior and six senior) but did not include any potentially distracting environmental perception events (i.e., change of clock display, phone call, operating light switched off). However, the SA level 1 scores were still low (*M_junior_* = .53, *M_senior_* = .47). We pooled the data for further analysis.

The analysis of the global situation perception score, the environment perception, and SA level 1 score indicated no significant difference between junior and senior anesthesiologist (Table 3). Finally, the expert rating on the overall situation awareness of the patient based on all questions (including situation awareness levels 2 and 3) showed that 27% of the junior anesthesiologists and 26% of the senior anesthesiologists were aware of the patient status.

**Table 3:** Descriptive and Interferential Statistic for Different Perception and Situation Awareness Scores and Model Fits for Junior and Senior Anesthesiologist

Variable	Junior (*n* = 30)	Senior (*n* = 31)	Test Statistics
Global situation perception	0.50 (0.15, 0.25–0.73)	0.56 (0.18, 0.42–0.75)	*t*(48) =1.589, *p* = .119, *d* = .36
Environmental situation perception	0.48 (0.15, 0.29–0.83)	0.56 (0.18, 0.14–0.86)	*t*(48) =1.578, *p* = .121, *d* = .48
Situation awareness level 1	0.53 (0.17, 0.20–0.80)	0.55 (0.14, 0.40–0.80)	*t*(59) =.384, *p* = .703, *d* = .13
Model fit phase 1–5	0.725 (0.077, 0.551–0.829)	0.718 (0.064, 0.571–0.830)	*t*(59) =.356, *p* = .723, *d* = .01
Model fit phase 5 laryngoscopy	0.821 (0.017, 0.799–0.882)	0.824 (0.022, 0.799–0.886)	*t*(59) =.461, *p* = .647, *d* = .15
Overall situation awareness	27% 8 out of 30	26% 8 out of 31	Fischer Exact test, *p* = 1

*Note.* For model fit, the descriptive data represents the summary of individual model fits (i.e., correlation of empirical PDT and predicted PDT for each participant) for the respective groups. Values indicate *M* (*SD*, range) or frequencies. The data of 11 participants did not include any environmental events (see Perception and Situation Awareness Scores Section).

### SEEV Model Fits

**Figure 1:**
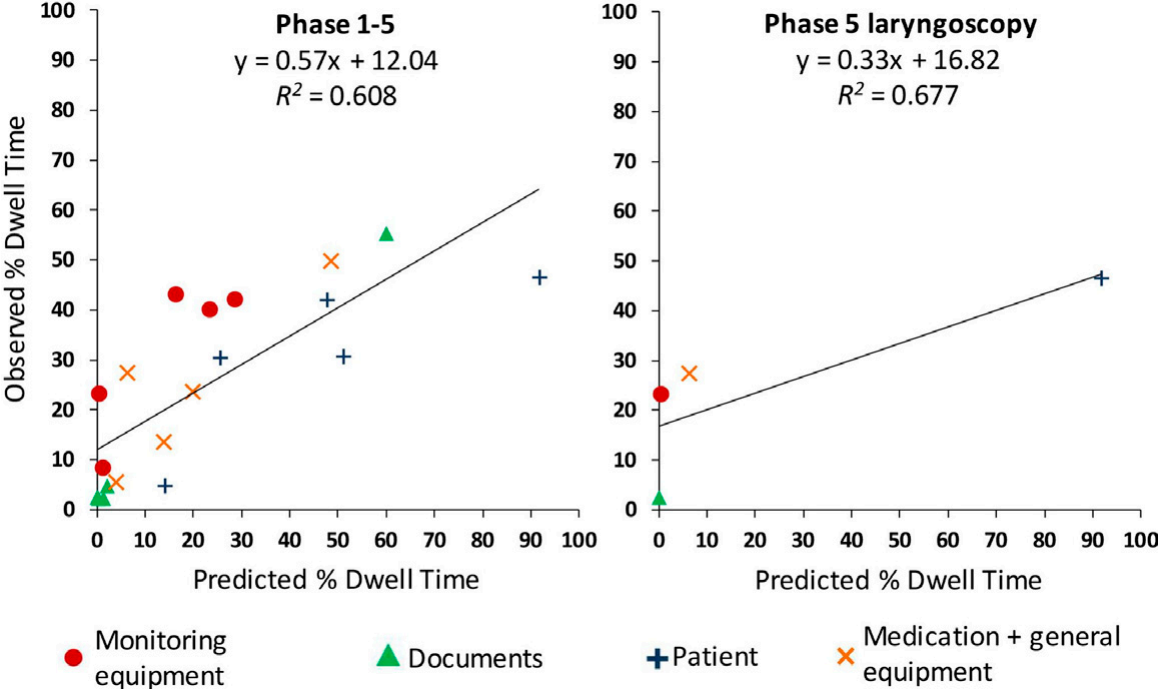
Scatterplot of predicted and average observed percent dwell time for all phases (left panel) and only phase 5 laryngoscopy (right panel).

Table 2 shows the empirical dwell times on the different AOI groups. Overall, 91.15% of the dwell time was considered in the EV model analysis. The correlation of the overall mean observed PDT of all participants and the predicted PDT based on 20 data points (5 phases × 4 AOI groups) showed a significant positive correlation of *r* = .780, *p* < .001 (Figure 1, left panel).

We calculated the model fit for each participant by correlation the observed PDT and the predicted PDT and used these correlations to compare the model fit of junior and senior anesthesiologists. Assessing the individual model fits for all phases showed no significant difference between junior and senior anesthesiologists (Table 3). Considering only the laryngoscopy (phase 5), the overall mean observed PDT of all participants and predicted PDT showed a nonsignificant positive correlation based on 4 data points (1 phase × 4 AOI groups) of *r* = .823, *p* = .177 (Figure 1, right panel). Again, assessing the individual model fits for the laryngoscopy showed no significant difference between junior and senior anesthesiologists (Table 3).

### Perception and Situation Awareness Scores, SEEV Model Fit, and PDT Correlations

We correlated the individual model fit for each participant for all phases (1–5) and for phase 5 (laryngoscopy) with the global situation perception, SA level 1, and environment perception scores (Table 4). We observed a small significant positive correlation of *r_s_* = .293 for the overall model fit (phase 1–5) and global situation perception and a small significant positive correlation of *r_s_* = .255 for model fit for the laryngoscopy (phase 5) and SA level 1.

**Table 4:** Spearman-Rho Correlations (*p*-Values) Between Model Fits for All Phases (1–5), Only the Laryngoscopy (Phase 5), the Observed Percentage Dwell Time (PDT) for the Different Area of Interests (AOI) Groups, and Different the Perception and Situation Awareness Scores

	Global Situation Perception (*n* = 50)	Situation Awareness Level 1 (*n* = 61)	Environment Perception (*n* = 50)
Model fit phase 1–5	**0.293** (.**039**)	0.217 (.094)	0.159 (.270)
Model fit phase 5	0.154 (.285)	**0.255** (.**047**)	0.059 (.685)
PDT AOI group *patient* phase 5	−0.341 (.**015**)	0.199 (.123)	−0.322 (.**022**)
PDT AOI group *monitoring equipment* phase 5	**0.336** (.**017**)	**0.257** (.**046**)	**0.351** (.**013**)
PDT AOI group *medication +general equipment* phase 5	0.234 (.102)	0.201 (.120)	0.178 (.216)
PDT AOI group *not included in EV analysis* phase 5	**0.416** (.**003**)	0.093 (.477)	**0.382** (.**006**)

*Note.* The data of 11 participants did not include any environmental events. Therefore, the *n* was different for the situation awareness level 1 correlations. The PDT of the AOI group documentation was zero in phase 5. Significant correlations (*p* < .05) are in bold font.

Finally, we used the expert categorization of participants regarding overall situation awareness based on the answers to all situation awareness questions (including the final two open questions regarding situation awareness levels 2 and 3) to compare model fit between these groups. We compared differences in model fit between the participants (*n* = 16) who were scored to have overall situation awareness and the other participants (*n* = 45). Against our expectations, we observed no significant difference for overall model fit (phase 1–5) between the group with overall situation awareness (*M* = .731, *SD* = .052) and without overall situation awareness (*M* = .718, *SD* = .076), *t*(59) = 0.630, *p* = .531, Hedges*’ g* = .18. Similarly, for laryngoscopy model fit (phase 5), we observed no significant difference between the group with overall situation awareness (*M* = .829, *SD* = .024) and without overall situation awareness (*M* = .821, *SD* = .014), *t*(59) = 1.417, *p* = .162, Hedges*’ g* = .47.

To complement our analysis, we also investigated the association of observed PDTs in phase 5 with the perception scores, as in previous research (Law et al., 2020; Moore & Gugerty, 2010). As summarized in Table 4, the PDT on the AOI group patient showed significant negative correlations with global situation perception (*r_s_* = −.341) and environmental perception (*r_s_* = −.322). The PDT on the AOI group monitoring equipment showed significant positive correlations with global situation perception (*r_s_* = .336), SA level 1 (*r_s_* = .257), and environmental perception (*r_s_* = .351). The PDT on the AOI not included in the EV analysis showed a significant positive correlation with global situation perception (*r_s_* = .416) and environmental perception (*r_s_* = .382). Finally, the observed PDT on the AOI group was zero in phase 5.

## Discussion

Overall, the hypotheses based on the Attention-Situation Awareness model (Wickens et al., 2008) were supported. Considering all participants, the overall model fit (phases 1–5) showed generally positive correlations with SA level 1 and the environmental perception scores and a significant but small correlation with global situation perception (*r_s_* = .293). These results may be considered as support for the idea that situation perception builds up (or decreases) based on the preceding attention allocation (Wickens et al., 2008). That is, participants who showed a better overall model fit built up situation perception in all phases of the induction and therefore showed increased scores in the situation perception scores. However, to fully support this explanation, the comparison of participants who were scored by the blinded domain expert to have overall situation awareness (levels 1–3) and the other participants should have shown a difference in overall model fit (phases 1–5).

The significant correlation of model fit during the laryngoscopy (phase 5) and SA level 1 supports the idea that the SEEV model can be used as a process situation awareness measure for level 1. Good attention distribution, according to the EV model fit in the phase where all events happened, was associated with higher SA level 1 scores. However, the correlation was small (*r_s_* = .255). This may be due to the fact that the SEEV model only addresses visual attention, and one may argue that two of our clinical events (the drop in heart rate and ventilator alarm) might have been primarily noticed via the sound of a dropping heart rate and an alarm sound. Furthermore, due to the experimental design in the present study, situation awareness was only assessed in one specific phase and with one SAGAT probe. Several SAGAT probes in various phases of a scenario would have resulted in more robust estimates for situation awareness scores and would have enabled an association between SEEV model fit and situation awareness scores for various phases.

For the model fit during the laryngoscopy (phase 5), we observed no significant difference between participants who were scored to have overall situation awareness (levels 1–3) and the other participants. However, the descriptive difference was in the expected direction, and the effect size was close to being medium (Hedges*’ g* = .47). Furthermore, in the Attention-Situation Awareness model (Wickens et al., 2008), the visual attention distribution is specifically considered as the SA level 1 process measure (the attention model) and is not the only factor influencing the overall situation awareness (the belief model). For example, in the present study, the anesthesiologists had expectations about the status of the patient based on previous phases (i.e., outdated but not necessarily wrong information) and pre-existing response strategies based on anesthesiologists’ general experience of inductions of general anesthesia. Direct situation awareness measures of expert participants in a representative task will always be influenced by participants’ pre-existing response strategies, which may increase the likelihood of error but may also be correct in many situations. Future research in various safety-critical domains should aim at implementing the full Attention-Situation Awareness model to be able to make better predictions about the belief module (i.e., situation awareness levels 2 and 3). Finally, even though the senior anesthesiologists had more work experience and a higher qualification, the junior anesthesiologists were fully qualified physicians and had knowledge and training in managing an induction of general anesthesia. This is also indicated by the similar and, in general, good model fit for the laryngoscopy (junior *M* = .821; senior *M* = .824). For the correlation of situation awareness and model fit, such a restricted range in one variable poses a methodological problem. Future research addressing experience may consider including final-year students in the sample (Hogan et al., 2006) or consider a more challenging scenario (Schulz et al., 2011) to increase the variability due to experience.

The results support the validity of the EV model. First, the overall model fit (phases 1–5) was acceptable with an *r* of 0.780. Compared to a previous study on the induction of general anesthesia (*r* = .845, Grundgeiger et al., 2020b), the current model fit was worse. One difference between the studies is that we did not include the final phase of the induction (mechanical ventilation and maintenance of general anesthesia) because we aborted the induction after the endotracheal intubation to conduct the situation awareness questionnaire. Like in a previous study (Grundgeiger et al., 2020b), we observed no difference in model fit for junior and senior anesthesiologists. This may be due to the common and uneventful scenario (i.e., a scenario without any critical incidence but an uneventful induction of general anesthesia). Previous research has shown differences in attention allocation between junior and senior anesthesiologists only in scenarios with a critical incident, such as a severe anaphylactic reaction, and not in uneventful scenarios (Schulz et al., 2011). Second, as discussed in the introduction, the EV model is supposed to assess “optimal” attention distribution and represents a gold standard against which the observed PDT can be compared (Wickens et al., 2008). Our results further support the idea of an “optimal” attention distribution by showing a positive correlation between the EV model fit and SA level 1.

Finally, we conducted a computational simpler analysis by correlating the observed PDT on the different AOI groups with the perception scores. The AOI patient indicated significant negative correlations between the PDT and global perception scores and environment perception scores. The same significant but this time positive correlations were observed for the AOI not included in the EV analysis. These correlations make sense considering that the environment perception events were not related to the AOI patient but may have been noticed when looking around the room. The positive significant correlations of the AOI monitoring equipment with SA level 1 makes sense because the content of the SA level 1 questions were present in the monitoring equipment. However, the significant correlations with global perception scores and environment perception scores were also positive and descriptively even larger than the correlation to monitoring equipment. Similar to the negative correlations of situation awareness and PDT on the supposing important AOI in a previous study Hasanzadeh et al. (2016), the latter correlations are difficult to explain.

The present SEEV model findings support previous research investigating the association of eye tracking metrics and situation awareness measures (Law et al., 2020; Moore & Gugerty, 2010). Similar to Moore and Gugerty (2010), SA level 1 was associated with model fit but, also similar to Moore and Gugerty (2010), the explained variance was small. One may question whether the more complex model analysis adds value. In our view, the SEEV model has several advantages. First, as explained in the introduction, the model can consider several AOI in one single analysis and, therefore, situation awareness questions are not limited to single AOI. Second, the model calculations are more complex compared to a pure correlation analysis but as a result provide a simpler final analysis. Based on the above discussion, we consider the EV model analysis also to be more cogent than the correlation analysis. Third, the theoretical foundation of the model and the a priori parametrization of the model factors enable specific predictions, such as PDT for specific AOI, and more general predictions including several AOI compared to a correlational analysis. Similar to the so-called Noticing-SEEV model that can be used to predict miss rates and response times (Steelman et al., 2013), the SEEV and the full Attention-Situation Awareness model may be used to predict situation awareness.

The study has several limitations. First, our study was powered only to detect a large experience effect (*f* = .40) with 1-β =.80. Research has shown that experience affects situation awareness (e.g., Endsley, 2006; Hogan et al., 2006). During laryngoscopy, experience may have a smaller effect on situation awareness than we could have detected with our sample size. Second, our situation awareness measure was based only on a single SAGAT probe (and not several measurement time points, see Wright et al., 2004). For example, Endsley (2000) recommends 30–60 probes. More probes would have provided situation awareness estimates that are more reliable. However, based on the present research question, we did not see another possible approach to assess situation awareness. Third, our probe included only a limited number of questions. More items would have provided a more sensitive measure. However, the number of reasonable questions for the present procedure is limited (Dishman et al., 2020), and a recent review on situation awareness measures shows that other healthcare studies using the SAGAT have used a similar number of items (Endsley, 2021). Fourth, we did not conduct a goal directed task analysis to design the SAGAT items (Wright et al., 2004). However, our identified goals and SAGAT questions are in line with the goals and SAGAT items for the induction of general anesthesia that have been recently identified by Dishman et al. (2020), using a goal directed task analysis.

## Conclusion

The results support the suggestion that the SEEV model fit can be considered a process measure of SA level 1 and that the EV model fit represents “optimal” scanning. If future research with more sensitive SA measures confirms this finding, the SEEV model may be a suitable method to analyze eye tracking data beyond the descriptive level and to be used as an estimate for SA level 1. Building on this finding and similar to the suggestions of de Winter et al. (2019), future applications of the SEEV model in combination with the Attention-Situation Awareness model may be able to predict situation awareness for specific time points. Such information could be used for training or, if the analyses could be done in real time, the design of interfaces that may increase the salience of specific information that does not receive enough attention. Furthermore, it appears that during the brief phase from the insertion until the removal of the laryngoscope blade, anesthesiologists do not have the capacity to also perceive and understand clinically relevant elements in the environment. Critically, this includes a sudden drop in the patient’s heart rate from 74 to 51 bpm, which was noticed by only 13 of 61 participants. Work place and technology designers should be aware that these situations can occur, and they should consider technology design, such as more salient information presentation or changed work practices, to empower operators to ask attending staff or additional staff for support.

## Key Points

- Eye tracking metrics have frequently been suggested as process situation awareness measure for safety-critical workspaces, but little research has investigated their association empirically and previous research was limited to the analysis of a single area of interest.
- In the context of anesthesiologists inducing general anesthesia into a simulated patient, we showed that the SEEV model fit was correlated with situation awareness level 1 (i.e., perceiving changes in the environment). Therefore, the SEEV model fit can be considered a valid process situation awareness measure.
- During the endotracheal intubation, the situation awareness of anesthesiologists was generally low, even for clinically relevant events.

## Supplemental Material

Supplementary Material 1 - Supplemental material for The Validity of the SEEV Model as a Process Measure of Situation Awareness: The Example of a Simulated Endotracheal Intubation Click here for additional data file. Supplemental material, Supplementary Material 1, for The Validity of the SEEV Model as a Process Measure of Situation Awareness: The Example of a Simulated Endotracheal Intubation by Tobias Grundgeiger, Anna Hohm, Annabell Michalek, Timo Egenolf, Christian Markus and Oliver Happel in Human Factors: The Journal of Human Factors and Ergonomics Society
