# Supplementary material for: The Validity of the SEEV Model as a Process Measure of Situation Awareness: The Example of a Simulated Endotracheal Intubation
Source: Hum Factors. 2021 Feb 17;64(7):1181–94. doi: 10.1177/0018720821991651 (PMC9574898; doi:10.1177/0018720821991651)
Supplement: Supplementary Material 1 - Supplemental material for The Validity of the SEEV Model as a Process Measure of Situation Awareness: The Example of a Simulated Endotracheal Intubation [file sj-docx-1-hfs-10.1177_0018720821991651.docx]

SUPPLEMENTAL MATERIAL

**Details of the Procedure and Material**

- Participants were told to imagine that they were about to induce general anesthesia in their first patient of a regular working day. As in the real induction room in the hospital under study, upon entering the simulation room, an anesthetic nurse (actor) had already prepared the patient for the procedure. The nurse was unknown to all participants, and the same nurse participated throughout the experiment.
- After the last drug was administered by the nurse, the nurse walked to the equipment trolley behind the participant to pick up the equipment for endotracheal intubation (laryngoscopy device; endotracheal tube; syringe for blocking the tube, i.e. inflate a little balloon on the outside of the tube to prevent any air “leaking” out of the lungs). The nurse put a watch on his left wrist and put the syringe used to block the airway in his pocket of the gown. The nurse took the laryngoscopy device in the right hand and the endotracheal tube in the left hand. When the participant indicated to start the laryngoscopy (Phase 5), the nurse walked to the left side (from the participant) of the patient at about thorax height.
- When the participant placed the oxygen mask on the side to start the laryngoscopy, several events were triggered that were used to assess awareness. First, the experimenter in the control room called the nurse. The nurse passed the laryngoscopy device to the participant, took the phone out of the pocket of the gown, and answered the phone call by placing the phone at the right ear (squeezing the phone in between the shoulder and the ear). Second, as soon as the participant received the device, the wall-mounted clock (shown on a large LCD display) changed from an analog to a digital display and the heart rate of the patient dropped within six seconds from 74 to 51 bpm (just above the pre-set alarm threshold of 50 bpm). The nurse was instructed to wait for five seconds after picking up the phone and then say, “Late shift next Thursday? I am sorry but I cannot take that shift.” Third, as soon as the participant took the endotracheal tube, one of the operating lights (which was pointing at an IV line in the right area of the simulator) was switched off and the power supply of the anesthetic machine was cut, resulting in a constant visual and auditory warning (for a visualization of the setup, see supplemental materials Figure S1). At about this point, the nurse finished the phone call and placed the phone back in the pocket. The nurse connected the syringe to the cuff of the endotracheal tube and blocked it.

*Figure S1.* Staged photo for illustration purposes of simulation room setup. Nurse (left) passes endotracheal tube to the anesthesiologist who is performing the laryngoscopy with a video-based device (right; not wearing the eye tracker). Boxes and arrows indicate the location of the various events. Question 6 addressed the content of the phone call and is not visualized.

*Figure S2.* Staged photo for illustration of the four areas of interest (AOI) groups and single areas of interest:

- AOI group patient included patient’s head, patient’s thorax, IV access, face mask (Phase 3-5), patient’s mouth, video monitor of laryngoscope (Phase 5), and patient’s arm.
- AOI group monitoring equipment included anesthesia machine, patient monitor, changing settings on patient monitor, changing settings on anesthesia machine, and clock.
- AOI group documentation included patient record and anesthesia chart.
- AOI medication + general equipment group included nurse’s hands, infusion, face mask (Phase 1-2), respiratory tube, application of drugs, anesthesia trolley, video monitor of laryngoscope (Phase 1-4), and monitoring cable.
- Not included in the analysis were nurse’s head, fixation due to movement in room, floor, walls, content of front pocket, IV pumps not in use, desk, entrance door to simulation, and not classified (i.e., wall, sink, etc.).

*Table S1*

*Model parameter values. A human factors researcher (TG) and a domain expert (OH) established (1) the expectancy (bandwidth) of the four AOI groups during each phase, (2) the priority of the two goals during each phase, and (3) the relevance of the four AOI groups during each phase for each goal. Two further domain experts additionally assigned the above values, and we used the average of the three parameter sets rounded to the full integer as final model parameters. The three sets showed a significant correlation (r = 0.692; all p-values < 0.001) and the correlation between the final parameter set and the three individual parameter sets was, on average, r = 0.873*

|  | | | AOI groups | | | |  |
| --- | --- | --- | --- | --- | --- | --- | --- |
| Parameter | | Goal | Patient | Monitoring equipment | Documents | Medication + general equipment |  |
| Expectancy | |  |  |  |  |  |  |
|  | Phase 1 |  | 3 | 1 | 4 | 1 |  |
|  | Phase 2 |  | 1 | 4 | 1 | 4 |  |
|  | Phase 3 |  | 3 | 4 | 1 | 3 |  |
|  | Phase 4 |  | 4 | 4 | 1 | 2 |  |
|  | Phase 5 |  | 4 | 2 | 1 | 3 |  |
|  | Phase 6 |  | 2 | 4 | 2 | 2 |  |
| Relevance | |  |  |  |  |  | Priority |
|  | Phase 1 | Preparing patient | 3 | 3 | 5 | 2 | 1 |
|  |  | Maintaining stability | 4 | 3 | 5 | 2 | 2 |
|  | Phase 2 | Preparing patient | 2 | 4 | 2 | 5 | 2 |
|  |  | Maintaining stability | 2 | 4 | 2 | 5 | 1 |
|  | Phase 3 | Preparing patient | 5 | 4 | 1 | 4 | 2 |
|  |  | Maintaining stability | 4 | 5 | 1 | 3 | 3 |
|  | Phase 4 | Preparing patient | 4 | 4 | 1 | 3 | 2 |
|  |  | Maintaining stability | 5 | 5 | 1 | 3 | 3 |
|  | Phase 5 | Preparing patient | 4 | 4 | 1 | 3 | 2 |
|  |  | Maintaining stability | 5 | 5 | 1 | 4 | 3 |

*Note.* Phase 1 = case history; Phase 2 = pre-check; Phase 3 = preoxygenation and induction; Phase 4 = mask ventilation and administration of neuromuscular blocking drugs; Phase 5 = laryngoscopy including placement of the tracheal tube.

*Table S2*

## *EV Model Predictions for Percentage Dwell Time (PDT).* *We determined the value parameter for each AOI group by summing up the products of priority and relevance for each goal (preparing the patient, maintaining stability). Next, the expectancy and value parameters were normalized to avoid biasing the results. Subsequently, we calculated the attentional attractiveness for each of the four AOI groups in each of the five phases by multiplying the corresponding expectancy and value scores. Finally, we summed up the attentional attractiveness for each phase and calculated the predicted PDT for the different AOI groups. The model predicts the percentage of visual attention that should be spent on each of the four AOI groups in each of the five phases of the induction of general anesthesia.*

| Phase | Area of interest | Percentage dwell time  model prediction |
| --- | --- | --- |
| Phase 1  case history | Patient | 30.56 |
|  | Monitoring equipment | 8.33 |
|  | Documentation | 55.56 |
|  | Medication + general equipment | 5.56 |
| Phase 2  pre-check | Patient | 5.00 |
|  | Monitoring equipment | 40.00 |
|  | Documentation | 5.00 |
|  | Medication + general equipment | 50.00 |
| Phase 3  preoxygenation and induction | Patient | 30.84 |
|  | Monitoring equipment | 42.99 |
|  | Documentation | 2.34 |
|  | Medication + general equipment | 23.83 |
| Phase 4  mask ventilation and relaxation | Patient | 42.01 |
|  | Monitoring equipment | 42.01 |
|  | Documentation | 2.28 |
|  | Medication + general equipment | 13.70 |
| Phase 5  laryngoscopy | Patient | 46.70 |
|  | Monitoring equipment | 23.35 |
|  | Documentation | 2.54 |
|  | Medication + general equipment | 27.41 |

Note. We multiplied the expectancy and value scores to calculate the attentional attractiveness for each AOI group. Wickens et al. (2008) suggested a modification to the multiplicative model, in which expectancy and value are added up. The additive model produced a better fit with the data from pilots, and Wickens et al. (2015; 2008) argued that it is sensible to assume that the attentional attractiveness should not be zero, because pilots may check a display just to ensure that nothing has changed. However, as in a previous study in anesthesiology (Grundgeiger, Wurmb, & Happel, 2020), the additive model did not fit the present data better (r_additive_ = 0.734 vs. r_multiplicative_ = 0.780), and some AOI such as paper-based documentation could not change without human action. We therefore conducted our analysis with the multiplicative model.
